# RETRACTED ARTICLE: Severe hyperpigmentation and scarring following glycolic acid peel treatment in combination with low-dose isotretinoin

**DOI:** 10.1186/s40001-014-0060-x

**Published:** 2014-11-07

**Authors:** Peter Arne Gerber, Gabriela Kukova, Edwin Bölke, Bernhard Homey, Evelyn Diedrichson

**Affiliations:** 1 Department of Dermatology, Medical Faculty, Heinrich Heine University Dusseldorf, Moorenstrasse 5, Duesseldorf, 40225 Germany; 2 Department of Radiation Oncology, Medical Faculty, Heinrich Heine University Dusseldorf, Moorenstrasse 5, Duesseldorf, 40225 Germany

The Editor-in-Chief has retracted and removed this article. After publication, concerns were raised about the anonymisation used in the images of the patient in this case study. Attempts to contact the paper’s authors to correct this issue were unsuccessful. As such, the article has been removed to protect patient’s privacy.

None of the authors responded to correspondence about this retraction.

